# Reply to the Letter to the Editor referring to “Dual-energy CT for the detection of skull base invasion in nasopharyngeal carcinoma: comparison of simulated single-energy CT and MRI”

**DOI:** 10.1186/s13244-023-01534-2

**Published:** 2023-12-18

**Authors:** Yang Zhan, Peng Wang, Yuzhe Wang, Yin Wang, Zuohua Tang

**Affiliations:** 1 Shanghai Institute of Medical Imaging, Fudan University, Shanghai, 200032 China; 2 Department of Radiology, Eye & ENT Hospital of Shanghai Medical School, Fudan University, 83 Fenyang Road, Shanghai, 200031 China; 3 Department of Radiology, Affiliated Hospital of Jiangnan University, Wuxi, 214122 China

**Keywords:** Dual-energy CT, Nasopharyngeal carcinoma, Skull base invasion

Dear Editor-in-Chief,

We are grateful for the author’s comments [1] on our article entitled “Dual-energy CT for the detection of skull base invasion in nasopharyngeal carcinoma: comparison of simulated single-energy CT and MRI” [2] and would like to take the opportunity to answer his interesting comments.

DECT is a functional imaging technique generated from two different energy datasets; it can use an image-based algorithm to reconstruct a series of material density maps to selectively display specific materials such as iodine [3, 4]. Due to the similar attenuation characteristics of iodine and calcium at low kVp, the calcium-containing voxels may be as bright as the iodine content on the iodine map [4]. However, in sclerotic lesions, the invasion of bone by tumor was associated with pathologic condition of bone metabolism and osteoblastic activity, leading to vascular network forming [5] and deposition of new bone [6], which were characterized by higher attenuation (iodine and bone content) in sclerotic lesion than normal bone on iodine map. Thus, the distinction between sclerotic/osteolytic and normal bones is reasonable and our DECT results are reliable as shown in our article.

For nasopharyngeal carcinoma patients, chemotherapy combined with radiotherapy is recommended for treating locoregionally advanced disease [7]; therefore, it is difficult to obtain the bone tissues in practice. In our study, the reference standard of skull base invasion was based on a combination of imaging features of all imaging techniques and 6-month follow-up, which was consistent with a previous study [8] and was regarded as an applicable clinical standard. Thus, these criteria for the standard reference were reliable and reasonable. Moreover, after 6-month follow-up of nasopharyngeal carcinoma patients, the lesions that did not change and were considered to be tumor invasions were due to the effect of simultaneous chemotherapy/radiotherapy.

The score of 4 (probably positive) in the imaging evaluation section of our manuscript was defined as invasion passing through one side of the bone cortex but not through the other side (penetration) of the bone cortex [9, 10].

Sincerely,

Zuohua Tang et al.

## Data Availability

Not applicable.
